# Does bullying predict suicidal behaviors among in-school adolescents? A cross-sectional finding from Tanzania as an example of a low-income country

**DOI:** 10.1186/s12888-019-2402-2

**Published:** 2019-12-16

**Authors:** Festo K. Shayo, Paul S. Lawala

**Affiliations:** 1 Department of Internal Medicine, Muhimbili University of Health and Allied Sciences, P. O Box, 65001 Dar es Salaam, Tanzania; 2 Department of Psychiatry and Mental Health, Mbeya Zonal Referral Hospital, P.O. Box 419, Mbeya, Tanzania; 3 Department of Global Health Entrepreneurship, Division of Public Health, Graduate School of Tokyo Medical and Dental University, 1-5-45 Yushima, Bunkyo-ku, Tokyo, 113-8510 Japan; 4 Dar es Salaam, Tanzania

**Keywords:** Adolescents, School bullying, Suicide behaviors, Tanzania, Low-income country

## Abstract

**Background:**

Bullying and suicidal behaviors are a silent public health problem among adolescents. Little is known about the link between bullying and suicidal behaviors in low-income countries such as Tanzania. In the current study, we estimated the prevalence of being bullied and determined its association with suicidal behaviors among in-school adolescents.

**Methods:**

We performed a secondary analysis of the Tanzania Global School-based Student Health Survey (GSHS) conducted in 2014. This was the first nationally representative survey conducted to a sample of 3793 in-school adolescents. The primary independent variable was being bullied, while the outcome variables of interest were suicide ideation and suicide attempt. We used a chi-square χ^2^ test for group variables comparisons and multivariate logistic regression for statistical associations between independent and outcome variables. In our analysis, a *p* < 0.05 was considered statistically significant at 95% confidence intervals.

**Results:**

The prevalence of being bullied among 3793 surveyed in-school adolescents was 27.0%. In an adjusted multivariate regression model, being bullied was independently associated with suicidal ideation and suicide attempt: [AOR; 1.9, 95% C.I; 1.5–2.4], and [AOR; 3.6, 95% C.I; 2.9–4.5] respectively, *p* < 0.001.

**Conclusions:**

Bullying is prevalent and possibly a potential predictor of suicidal behaviors among in-school adolescents in Tanzania. There is a need for all educational stakeholders: teachers, parents, students, mental health professionals, and policymakers to design a program for mitigating the problem of bullying in schools.

## Background

Bullying in schools has been defined as ‘unwanted, aggressive behavior among school-aged children. In due course of time, the behavior is likely repeated and involves a real or perceived imbalance of power [1]. Bullying in schools infringes on the rights of children and adolescents to education and health [2, 3]. It has short- and long-term negative impacts on adolescents’ development in terms of physical, social, and psychological well-being [4]. The victims of bullying usually experience a number of problems, such as anxiety and depression; suicide behaviors; poor school attendance and dropout; and poor academic achievement [4–6]. For instance, a study conducted in 42 high-income countries of Europe and North America found that bullying victims were more likely to have poor socioeconomic status, a difficult relationship, and psychiatric disorders [4]. School bullying is one of the silent public health concerns that need a dedicated team of families, educators, health-care professionals, and policy-makers to mitigate [4, 7].

In the Tanzania context, bullying (“being bullied”) is defined as an act of a person being picked on over and over again by an individual or group with more power, either in terms of physical strength or social status [3]. Few case studies conducted in Tanzania have reported the burden of bullying among in-school adolescents. In those studies, the most common type of bullying was physical bullying in the form of hitting, kicking, biting, spitting, pushing, and stilling and threatening using a weapon. Male students than female adolescents were more preferred bullies. Nevertheless, among reported effects of bullying were a poor academic performance, social-emotional disorders, and depression [2, 3].

Suicide is the major public health problem in the world accounting for about 6% of all deaths in young people [8]. There are four components of suicide behaviors, namely suicide ideation, a suicide plan, a suicide attempt, and committing suicide (killing oneself) [9]. Furthermore, bullying, violent behaviors, and psychiatric conditions are known as a risk factors for suicide behaviors among adolescents [10]. About 90% of children and young adults in the world live in low- and middle-income (LMICs): 75% of suicide mortality occurs in this region [11].

In 2012 Tanzania had twice as a higher suicide rate than the global average: 24.9 versus 11.4 per 100,000 populations. The suicide rate was 3.5 and 20.7 per 100,000 populations in the age groups 5–14 years and 15–29 years, respectively [9].

Studies in high-income countries have reported a link between bullying and suicidal behaviors among school adolescents. For instance, studies in different parts of the United States of America found that bullied students were significantly more likely to experience suicide ideation, planning, and attempt [12–14]. In Greece, a study found that being bullied was an independent predictor of suicide ideation among adolescents regardless of the presence of psychiatric disorders [15]. Moreover, a study in Korea showed that adolescents who experienced physical and non-physical bullying were significantly three times more likely to attempt suicide than their counterparts [16]. In Africa, two studies have reported the link between suicidal behaviors and school bullying. Studies in the Republic of Benin and Malawi reported that the following factors were associated with the likelihood of suicidal attempts among in-school adolescents; anxiety, loneliness, and being bullied [17, 18].

In Tanzania, information is limited about the national prevalence of bullying and its association with suicidal behaviors among in-school adolescents. A single small scale survey conducted among in-school adolescents in Dar es Salaam city in 2006 reported a prevalence of 7 and 6.3% for suicidal ideation and suicidal plan, respectively. It was revealed that psychological factors (loneliness and anxiety), and being bullied were among the factors significantly associated with suicidal behaviors [19]. Although the burden of school bullying in Tanzania has been reported by a few small scale studies, it is less emphasized and underestimated. Therefore, in the current study, we explored the national prevalence of school bullying and its association with suicidal behaviors among in-school adolescents.

## Method

### Data source

In the current study, we did a secondary analysis of the first Tanzania Global School-based Student Health Survey (GSHS) conducted in 2014. The 2014 GSHS was conducted by the Tanzania Ministry of Health and Social Welfare (MoHSW) in collaboration with the Ministry of Education and Vocational Training (MoEVT), under the National School Health Programme (NSHP). World Health Organization (WHO) and the Centre for Diseases Control (CDC) provided financial and technical assistance. The national GSHS Coordinator organized and supervised the survey with strict adherence to the standard GSHS methodology. The dataset for the current analysis was obtained from the WHO public domain: http://www.who.int/ncds/surveillance/gshs/tanzaniadataset/en/.

### Sampling, sample size, and data management

Tanzania 2014 GSHS used a two-stage cluster sampling to obtain a representative sample of students in primary schools (grade/standard 6–7) and in secondary schools (form 1–3), who corresponds with the GSHS targeted aged range 13–17 years. The school response rate was 100%, while the student response rate was 87%. Overall, 3797 of the 4373 sampled students completed questionnaires, and 3793 questionnaires were usable after data editing. More details about the methodology can be accessed in the preceded publications [20–22].

### Operational definitions

In the current study, we defined bullying as when a student or group of students say or do bad and unpleasant things to another student. It is also regarded as bullying when a student is teased a lot in an unpleasant way or when a student is left out of things on purpose [1, 3].

Suicide behaviors refer to a range of four components behaviors including suicide ideation, suicide planning, suicide attempt, and committing suicide (killing oneself) [13]. In the current study, we defined suicide behaviors based on two components: suicide ideation and suicide attempt.

### Study variables

#### Outcome variables

The primary outcome variables were the suicide ideation and suicide attempt. The study participants were categorized into two groups of those who reported suicidal ideation and those who reported a suicide attempt. The variables were obtained from the two questions response of the Tanzania GSHS questionnaire. For suicide ideation, the participants were asked: During the past 12 months, did you ever seriously consider attempting suicide? Responses were Yes and No. For a suicide attempt, the participants were asked: During the past 12 months, how many times did you actually attempt suicide? The response was Yes for 1,2,3,4,5, and ≥ 6 times attempts and No for zero attempts.

#### Independent variables

The primary independent variable was being bullied [3]. Participants were asked: During the past 30 days, on how many days were you bullied? The response ranged from zero, 1–2, 3–5, 6–9, 10–19, 20–29, and all 30 days. This was then decoded as Yes for 1 to all 30 days, and No for zero days. The other independent variables were loneliness and anxiety. Regarding loneliness, the participants were asked*:* During the past 12 months, how often have felt lonely? Responses: never, rarely, sometimes, most of the time, and always. The responses were categorized into Yes (combined most of the time and always) and No (never, rarely, and sometimes). For the Anxiety, the participants were asked: During the past 12 months, how often have you been so worried about something that you could not sleep at night? The responses were categorized into Yes (combined most of the time and always) and No (never, rarely, and sometimes). Also in the current analysis, we have included the following variables gender (male and female), age (categorized into ≤12 yrs., 13–17 yrs., and > 17 yrs), and school grade (categorized into primary and secondary school).

#### Data process and analysis

We assessed for the pattern of missing data in the GSHS data set and found that the missing data followed a random pattern. Therefore, we used a Full Conditional Specification (FCS) command of multiple imputations to account for the missing data. We found that there was no bias in the results between the imputed dataset and the original dataset: replacing the missing data with imputed data did not make a significant difference. We run five imputations to achieve an efficiency of > 97%. The use of multiple imputations has been reported by previous studies [17, 20–23].

In the current study, we used both descriptive and inferential statistics to present our results. We used a chi-square χ^2^ test for group variables comparisons and multivariate logistic regression for statistical associations between independent and outcome variables. In our analysis, a *p* < 0.05 was considered statistically significant at 95% confidence intervals. We used SPSS version 22 (SPSS, Chicago, IL) in the current analysis.

## Results

### Demographic characteristics and prevalence of bullying

Table 1 represents the demographic characteristics of study participants, prevalence of bullying, suicidal behaviors, and other risk factors. Of the 3793 in-school adolescents, the prevalence of being bullied was 27% while, 52.1% of the adolescents were females.

**Table 1:** Demographics, the prevalence of bullying and suicidal behaviors among in-school adolescent *N* = 3793]

Variables	Categories	n[%]
Demographics		
Gender	Male	1819 [47.9]
	Female	1974 [52.1]
Age	≤12 yrs.	681 [18.0]
	13–17 yrs.	2987 [78.7]
	> 17 yrs.	125 [3.3]
Academic level	Primary school	2085 [55.0]
	Secondary school	1703 [44.9]
	Other grade	5 [0.1]
Bullying	Yes	1023 [27.0]
	No	2770 [73.0]
Loneliness	Yes	283 [7.5]
	No	3510 [92.5]
Anxiety	Yes	237 [6.2]
	No	3556 [93.8]
Suicide ideation	Yes	536 [14.1]
	No	3257 [85.9]
Suicide attempt	Yes	422 [11.1]
	No	3371 [88.9]

### Bullying and suicide behaviors

Table 2 represents the relationship between suicidal behaviour and being bullied. A significantly large proportion of adolescents with suicidal ideation and attempt had ever been bullied compare to their counterparts: 220 (41.0%) vs. 803 (24.7%), and 238 (56.4%) vs. 785 (23.3%), respectively.

**Table 2:** Comparison between bullying and suicidal behaviors among in-school adolescents (*N* = 3793)

Variables	Categories	Suicide ideation			Suicide attempt		
		Yes n[%]	No n [%]	*P* value	Yes n [%]	No n [%]	*P* value
Demographics							
Gender	Male	271 [50.5]	1548 [47.5]	0.19	192 [45.4]	1627 [48.2]	0.36
	Female	265 [49.5]	1709 (52.5)		230 [54.6]	1744 [51.8]	
Age	≤12 yrs.	150 [27.6]	531 [16.4]	< 0.001	110 [25.6]	571 [17.0]	< 0.001
	13–17 yrs.	380 [69.8]	2607 [80.2]		311 [72.3]	2676 [79.6]	
	> 17 yrs.	14 [2.6]	111 [3.4]		9 [2.1]	116 [3.4]	
School grade ^§^*N* = 3748	Primary	315 [59.7]	1750 [54.3]	0.03	262 [63.1]	1803 [54.1]	< 0.001
	Secondary	213 [40.3]	1470 [45.7]		153 [36·9]	1530 [45.9]	
Bullied	Yes	220 [41.0]	803 [24.7]	< 0.001	238 [56.4]	785 [23.3]	< 0.001
	No	316 [59.0]	2454 [75.3]		184 [43.6]	2586 [76.7]	
Psychological							
Loneliness	Yes	63 [11.7]	220 [6.8]	< 0.001	74 [17.5]	209 [6.2]	< 0.001
	No	474 [88.3]	3036 [93.2]		348 [82.5]	3162 [93.8]	
Anxiety	Yes	65 [12.1]	172 [5.3]	< 0.001	79 [18.7]	158 [4.7]	< 0.001
	No	471 [87.9]	3085 [94.7]		343 [81.3]	3213 [95.3]	

^§^ other grades category was not included

### Factors associated with suicide behaviors

#### Bullying and suicide behaviors

After adjusting for the potential confounders in the regression model, being bullied remained a significant independent predictor of suicide ideation and attempt: [AOR; 1.9, 95% C.I; 1.5–2.4], and [AOR; 3.6, 95% C.I; 2.9–4.5], respectively. For more details, refer to Table 3.

**Table 3:** Multivariate logistic regression of the association between bullying and suicidal behaviors among adolescents (*N* = 3793)

Variables		Suicide ideation		Suicide attempt	
		OR [95% C.I]	AOR [95% C.I]	OR [95% C.I]	AOR (95% C.I)
Age (yrs) (Ref. > 17 yrs.)	≤12	2.2 [1.2–4.2] *p* = 0.01	2.8 [1.4–5.5] *P* = 0.003	2.4 [1.2–4.8] *p* = 0.02	2.5 [1.2–5.6] *p* = 0.02
	13–17	1.2 [0.7–2.1]	1.4 [0.8–2.6]	1.5 [0.7–2.9]	1.7 [0.8–3.6]
Age ^a^		0.6 [0.4–0.7] *p* < 0.001	0.5 [0.4–0.7] *p* < 0.001	0.6 [0.5–0.8] *p* < 0.001	0·7 [0.5–0.9] *p* = 0.002
School grade (Ref. Secondary)	Primary	1.3 [1.0–1.5] *p* = 0.03	0.9 [0.8–1.2]	1.4 [1.2–1.8] *p* < 0.001	1.1 [0.9–1.5]
Gender (Ref. Female)	Male	1.1 [0.9–1.4]	1.3 [1.0–1.6] *p* = 0.03	0.9 [0.7–1.1]	0.9 [0.8–1.2]
Bullied (Ref. No)	Yes	2.1 [1.7–2.6] *p* < 0.001	1.9 [1.5–2.4] *p* < 0.001	4.3 [3.4–5.3] *p* < 0.001	3.6 [2.9–4.5] *p* < 0.001
Loneness (Ref. No)	Yes	1.8 [1.3–2.5] *p* < 0.001	1.4 [1.0–2.0] *p* = 0.04	3.2 [2.4–4.3] *p* < 0.001	1.9 [1.3–2.6] *p* < 0.001
Anxiety (Ref. No)	Yes	2.5 [1.8–3.4] *p* < 0.001	1.9 [1.3–2.6] *p* < 0.001	4.7 [3.5–6.3] *p* < 0.001	2.8 [2.0–4.0] *p* < 0.001

^a^age as a continuous variable

#### Psychological factors and suicidal behaviors

Adolescents with psychosocial factors: anxiety and loneliness were more likely to have experienced suicidal ideation and attempt than their counterparts. For more details, refer to Table 3.

#### Demographics and suicide behaviors

Younger adolescents (≤12 yrs) were significantly more likely to have experienced suicidal ideation and attempts than their counterparts. For more details, refer to Table 3.

## Discussion

Our current study found that being bullied was an independent predictor of suicidal ideation and attempt among in-school adolescents. The findings are in support of the previous studies in high-income countries of North America, Europe, and Asia [11–16, 24]. The association between suicidal behaviors and other psychological factors such as loneliness and anxiety in the current study was also comparable to previous studies elsewhere [4, 11, 17, 18]. Also, in our current study, we adjusted bullying with other strong predictors of suicidal behaviors like psychological factors: loneliness and anxiety. The purpose was to see whether being bullied can independently predict the likelihood of suicidal behaviors. Most of the reviewed studies in high-income countries did not consider bullying as an independent predictor of suicidal behaviors, except for the study conducted in Greece [15]. Although psychiatric and psychosocial factors are well-known predictors of suicidal behaviors, our study finding emphasizes that bullying is also a potential predictor of suicidal behaviors among in-school adolescents.

Furthermore, the current study found that near one-third of the in-school adolescents were bullied. The frequency of being bullied ranged from one to more than twelve times in the past 12 months preceding the survey. Moreover, a significantly large proportion of adolescents with suicidal ideation and suicide attempt had ever been bullied, experienced loneliness, anxiety, and were in primary schools. The prevalence of bullying in the current study was lower than studies in North America but higher than studies in Korea and Greece. The differences in the prevalence and burden of bullying across countries have been reported in previous studies elsewhere [4, 5]. Despite those differences, the hypothesis that bullying is an independent predictor of suicidal behaviors remains indisputable. However, we need further evidence from longitudinal studies to explain the phenomena behind the causal relationship between bullying and suicidal behaviors.

The strength of the current study is based on the following aspects: To our knowledge, this is the first nationally representative study in Tanzania to report the association between bullying and suicidal behaviors in adolescents. Therefore, the current study gave valuable insight to inform the education stakeholders and policymakers about the burden of bullying among in-school adolescents. Moreover, it is a piece of add-on evidence that a burden of school bullying is also prevalent among in-school adolescents in low-income countries, such as Tanzania. Nevertheless, the current study was able to show the independent link between bullying and suicidal behaviors, which were less reported by the previous studies. On the other hand, we used the information of physical bullying available the GSHS dataset file hence reflecting the most form of bullying in the Tanzania context [2, 3]. Lastly, the current study used multiple imputations to account for non-response variables (missing data). The purpose was to avoid the possibility of bias findings, interpretation, and representativeness.

The current study had the following limitations: First, the variables suicide attempt and bullying had multiple responses ranging from a single to multiple times events. Therefore, the variation between participants who experienced single suicide events and those experienced multiple events, might not be captured in the current study. Second, we included only one aspect of bullying: being bullied. This is because of the GSHS dataset file does not contain other aspects of bullying such as perpetrator (bullying others) and victim-perpetrator (both being bullied and bullying others). Nevertheless, the dataset did not categorize the four types of bullying: verbal bullying, social bullying, physical bullying, and cyberbullying. Therefore, our current findings may not give the global status of bullying among in-school adolescents. Third, the use of a single item to represent a broad variable, for instance, anxiety, may not suffice to give an overall implication of our findings. Fourth, the current study included only school-going adolescents, hence the finding may not be generalized to all adolescents.

To our knowledge, we think that bullying prevention requires a joint effort from all education stakeholders including teachers, parents, students, school administrators, policymakers, and the whole society. Therefore, we recommend the following: First, educational stakeholders to design a bullying prevention program in order to reduce the burden of bullying behaviors at school. Second, the ministry of health should campaign about the effects of bullying at school and in the community and on how to mitigate the problem. Furthermore, the problem of surrounding suicidal behaviors happening in the context of bullying shouldn’t be made secret. Third, the ministries of Health and Education should collaborate to mitigate the school bullying by endorsing a political will towards the protection of the right for education to all students. Also, the introduction of a new subject about adolescent psychology and behaviors in primary and secondary schools may be helpful to both students and teachers. Lastly, all health professionals who encounter adolescents reporting about bullying should also be curious to know about any background clue of suicidal behaviors and vice versa.

## Conclusion

Similar to high-income countries, our study showed that being bullied is prevalent among in-school adolescents in Tanzania as an example of a low-income country. Moreover, being bullied may be a potential independent predictor of suicidal behaviors. Therefore, it is of paramount importance for educational stakeholders including teachers, parents, students, mental health professionals, and policymakers to design a bullying prevention program at the school level.

## Data Availability

The dataset files used for analysis in the current study are publicly free available in the WHO repository (http://www.who.int/ncds/surveillance/gshs/tanzaniadataset/en/).

## Abbreviations

AOR: Adjusted odds ratio
C.I: Confidence interval
CDC: Centre for Diseases Control
GSHS: Global School-based Student Health Survey
MoEVT: Ministry of Education and Vocational Training
MoHSW: Ministry of Health and Social Welfare
OR: Odds ratio
WHO: World Health Organization
